# Cavitary pulmonary tuberculosis with *Orientia tsutsugamushi* coinfection in a non-endemic region: a case report

**DOI:** 10.3389/fmed.2025.1692918

**Published:** 2025-12-03

**Authors:** Yuanjiang Zheng, Jiangyan Hou, Li Yang, Youjun Jiang, Shanyu Wang, Jianglin Yu, Xianwei Ye

**Affiliations:** 1 Department of Respiratory and Critical Care Medicine, Guizhou Provincial People’s Hospital, Guiyang, Guizhou, China; 2 Graduate School, Zunyi Medical University, Zunyi, Guizhou, China

**Keywords:** cavitary pulmonary tuberculosis, *Orientia tsutsugamushi*, coinfection, metagenomicnext-generation sequencing, non-endemic region, indirect immunofluorescence

## Abstract

**Introduction:**

Coinfection of pulmonary tuberculosis and scrub typhus caused by *Orientia tsutsugamushi* is exceptionally rare. Overlapping clinical and radiologic features, together with the frequent absence of clear epidemiologic clues, complicate timely diagnosis.

**Case presentation:**

A 57-year-old man residing in a non-endemic region presented with a left-sided cavitary lung lesion on imaging. Computed tomography (CT)-guided percutaneous lung biopsy, acid-fast bacillus staining, and *Mycobacterium tuberculosis* DNA PCR established the diagnosis of active cavitary pulmonary tuberculosis. Despite initiation of a standard first-line anti-tuberculosis regimen, high-grade fever persisted. Metagenomic next-generation sequencing (mNGS) of bronchoalveolar lavage fluid (BALF) detected *O. tsutsugamushi*, which was subsequently confirmed by a positive IgM indirect immunofluorescence assay (IFA). Doxycycline was added, leading to defervescence within 48 h and marked symptomatic improvement. On follow-up, chest CT demonstrated lesion absorption and cavity shrinkage, while new fibrotic changes emerged. The patient was started on maintenance pirfenidone and prescribed home oxygen therapy.

**Conclusion:**

In patients with pulmonary tuberculosis who exhibit persistent fever or suboptimal response despite appropriate therapy—and after excluding drug resistance—scrub typhus should be included in the differential diagnosis, even in non-endemic settings without a typical exposure history. Longitudinal imaging in this case also shows that irreversible structural remodeling may occur despite microbiologic control, underscoring the need to pair prompt pathogen-directed therapy with ongoing monitoring and early strategies to preserve lung function.

## 1 Introduction

Scrub typhus is an acute, mite-borne zoonosis caused by *Orientia tsutsugamushi*, transmitted by the bites of infected chigger larvae, and is widely endemic across the Asia–Pacific region (1). Its clinical manifestations are protean: while acute fever, rash, lymphadenopathy, and the pathognomonic eschar are classic, a substantial proportion of patients lack typical cutaneous signs, complicating recognition—particularly outside endemic areas (2, 3). The disease can involve multiple organ systems; respiratory involvement is not uncommon and ranges from mild bronchitis and interstitial pneumonia to acute respiratory distress syndrome (4).

Pulmonary tuberculosis, caused by *Mycobacterium tuberculosis*, remains a major global infectious disease (5). Tuberculosis shares overlapping features with scrub typhus—including fever, respiratory symptoms, and radiographic pulmonary infiltrates—which increases the risk of missed or delayed diagnosis in non-endemic settings (5, 6). Concomitant infection with *O. tsutsugamushi* and tuberculosis is rare, and data on clinical characteristics, outcomes, and longer-term pulmonary sequelae remain limited.

Here, we report a case of cavitary pulmonary tuberculosis with confirmed *O. tsutsugamushi* coinfection in a non-endemic region, highlighting diagnostic pitfalls and the potential for persistent structural lung injury despite microbiologic control.

## 2 Case presentation

**Figure 1:**
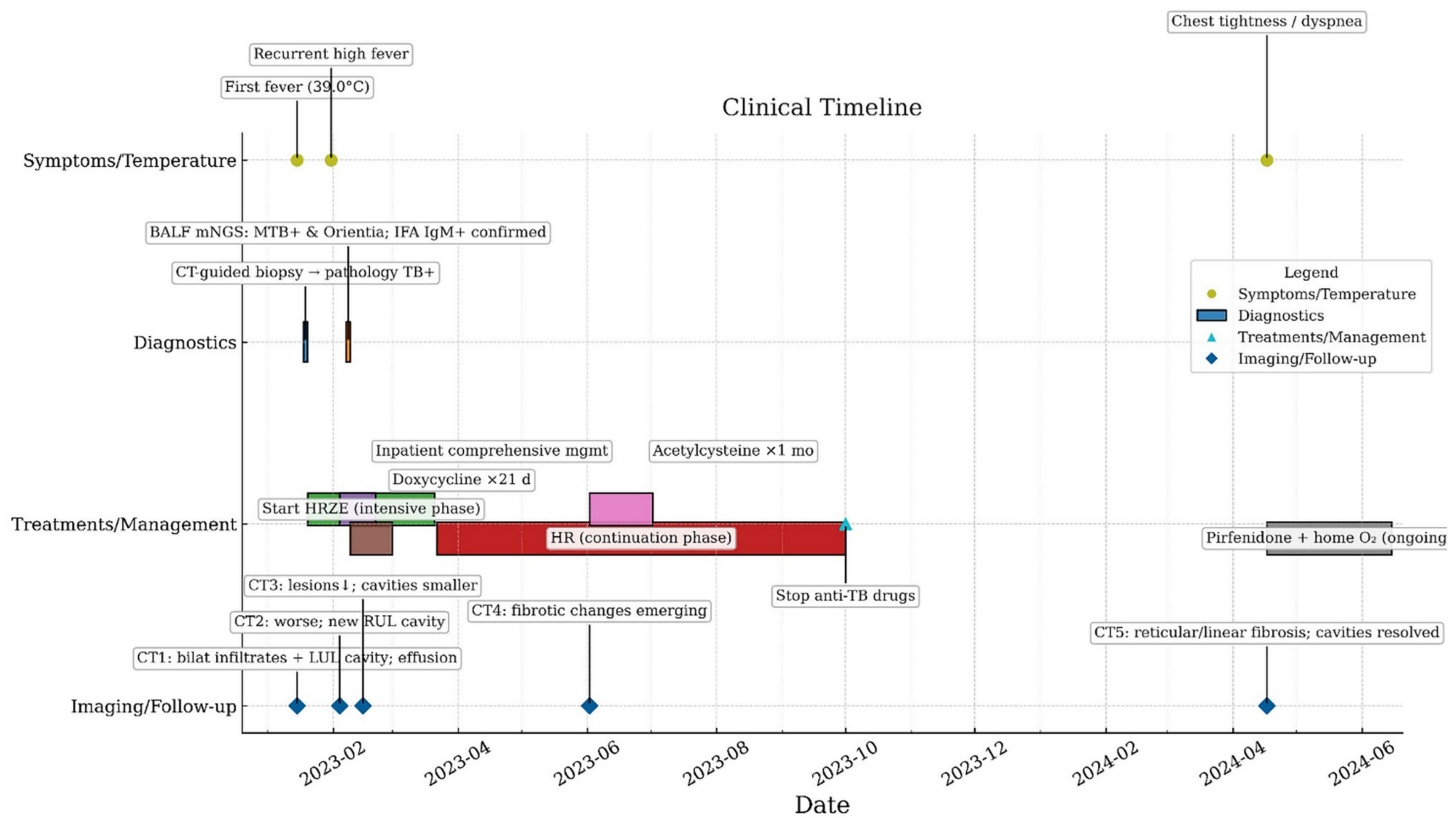
Patient treatment timeline.

**Figure 2:**
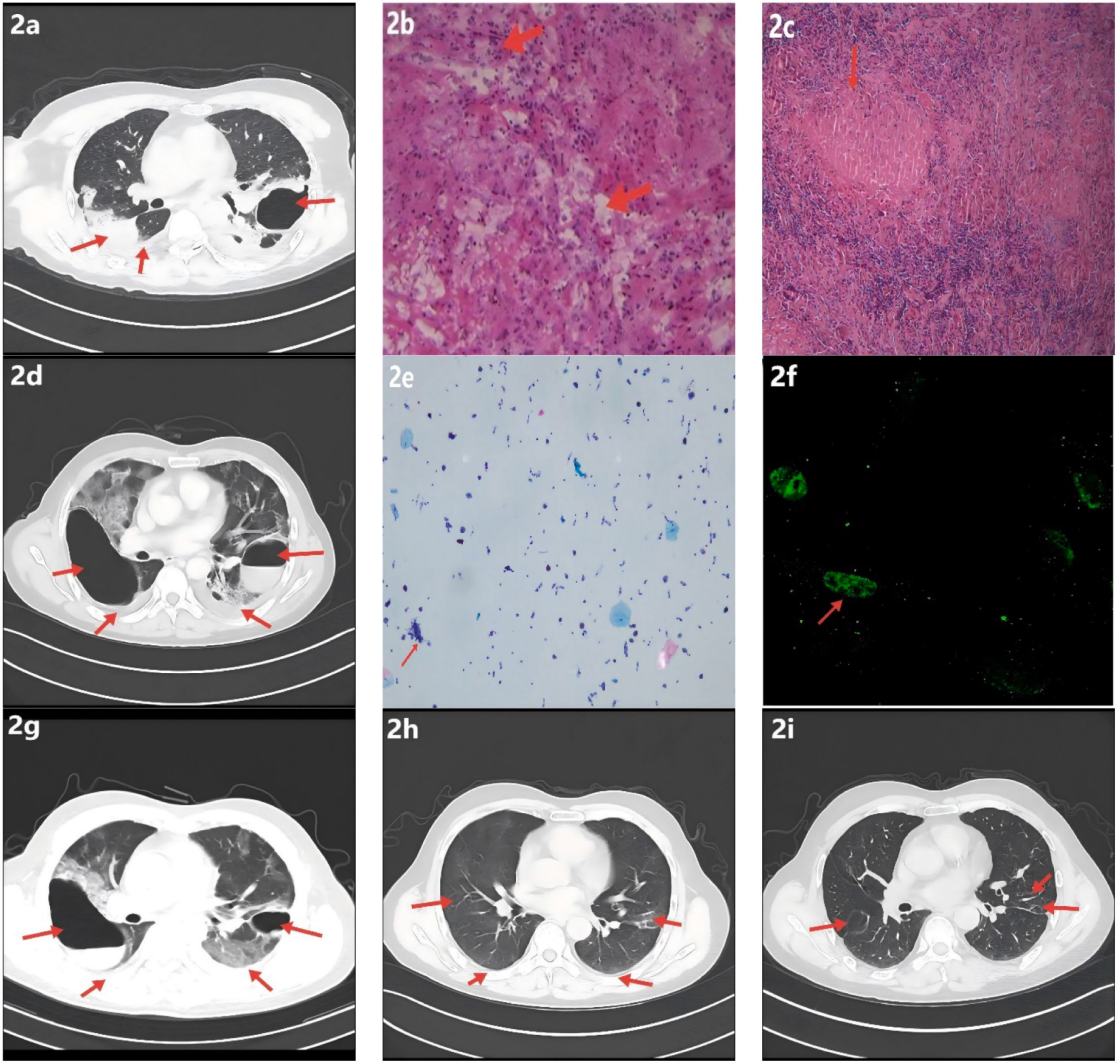
Radiologic, histopathologic, and laboratory findings. **(a)** Chest CT shows multifocal infectious opacities in both lungs with areas of consolidation and a cavitary lesion in the left upper lobe; small bilateral pleural effusions are present. **(b)** Hematoxylin–eosin staining demonstrates caseous necrosis surrounded by dense inflammatory cell infiltration. **(c)** Ziehl–Neelsen acid-fast bacillus stain is positive, supporting tuberculosis; Periodic acid–Schiff and Gomori methenamine silver stains are negative. **(d)** Interval CT reveals progression of multifocal pulmonary infection, a newly formed right upper-lobe cavity, slight enlargement of the pre-existing left upper-lobe cavity, and small bilateral pleural effusions (increased on the right, decreased on the left). **(e)** Bronchoalveolar lavage fluid differential cell count: epithelial cells ≈ 40% of total cells; inflammatory cells ≈ 60% (neutrophils ≈ 70%, lymphocytes ≈ 20%, histiocytes ≈ 10%). **(f)** Serum IgM IFA demonstrates bright, granular apple-green fluorescence; *Orientia tsutsugamushi* IgM is positive. **(g)** Follow-up CT shows decreased infectious lesions and reduction in the size of bilateral upper-lobe cavities; small bilateral pleural effusions with decreased volume on the right and a slight increase on the left. **(h)** Infectious opacities have nearly resolved, with marked contraction of upper-lobe cavities and near-complete absorption of bilateral pleural effusions; new focal fibrotic changes are evident. **(i)** Prior upper-lobe cavities have resolved, with bilateral focal reticular and linear fibrotic opacities.

A 57-year-old man was admitted on January 30, 2023, with a 1-month history of left-sided chest pain and cough producing scant white mucoid sputum, and a 15-day history of fever accompanied by hemoptysis (see Figure 1). One month prior to admission, he developed persistent dull pain over the left anterior chest without an identifiable trigger, along with cough and small amounts of whitish sputum but no hemoptysis; he did not seek formal medical care. On January 15, 2023, the patient developed fever (maximum 39.0 °C) accompanied by hemoptysis without clots. Chest CT at an outside hospital demonstrated multifocal bilateral infectious infiltrates with areas of consolidation, a thick-walled cavity with an air–fluid level in the left upper lobe, bilateral pleural effusions, and mildly enlarged mediastinal lymph nodes (Figure 2a). Based on the clinical and radiologic findings, mixed infection with Gram-negative bacilli and Gram-positive cocci was suspected. Empiric therapy with piperacillin–tazobactam (4.5 g every 8 h) plus ornidazole (0.5 g every 12 h) was initiated, together with nebulized N-acetylcysteine and salbutamol, Tanreqing injection for symptomatic relief, and therapeutic thoracentesis. Chest pain partially improved, but cough and sputum persisted. On January 18, 2023, a CT-guided percutaneous transthoracic needle biopsy was performed. Histopathology finalized on January 20 revealed granulomatous inflammation with caseous necrosis (Figure 2b). Ziehl–Neelsen acid-fast staining was positive (Figure 2c), and *Mycobacterium tuberculosis* DNA PCR was positive. Integrating the radiologic and pathologic findings, a diagnosis of active cavitary pulmonary tuberculosis was established. A guideline-concordant 2HRZE/4HR regimen—isoniazid 300 mg, rifampicin 600 mg, pyrazinamide 1,600 mg, and ethambutol 1,200 mg—was initiated and subsequently tailored according to serial sputum culture results and interval radiologic reassessments. The fever curve attenuated overall, yet intermittent fever persisted with a maximum temperature of 38.1 °C. For personal reasons, the patient was discharged on January 25 with instructions to continue the oral anti-tuberculosis regimen. On January 31, fever worsened (peak 39.6 °C) with ongoing cough, sputum, and hemoptysis; home ibuprofen provided inadequate control. He was readmitted on February 4, 2023, to our Department of Respiratory and Critical Care Medicine for further management. The patient was previously healthy, with no major chronic comorbidities. He had prior trauma to the scalp, right knee, and right chest, and denied residence in or travel to forested areas.

On admission, vital signs were: temperature 36.5 °C; respiratory rate 20 breaths/min; pulse 78 beats/min; blood pressure 124/67 mmHg; and peripheral oxygen saturation 98% while receiving 2 L/min supplemental oxygen via nasal cannula. The patient appeared subacutely ill, with an old scar at the vertex. Pulmonary examination revealed bilateral dullness to percussion with decreased breath sounds and a few moist crackles. Cardiac examination showed a regular rhythm at 78 beats/min. An old scar was present over the right knee, and there was no edema of either lower limb.

On the day of admission, laboratory testing showed elevated C-reactive protein (25.59 mg/L) and fibrinogen (6.17 g/L). Cardiac enzyme studies demonstrated increased lactate dehydrogenase (LDH, 264 U/L) and *α*-hydroxybutyrate dehydrogenase (α-HBDH, 213 U/L). Complete blood count revealed leukocytosis (11.09 × 10^9/L) with neutrophilia (absolute neutrophil count 8.22 × 10^9/L). Lymphocyte subset analysis and arterial blood gas analysis were unremarkable (see Supplementary Table 1). Contrast-enhanced chest CT with three-dimensional reconstruction indicated progression of multifocal pulmonary infection compared with prior imaging, with a newly formed right upper-lobe cavity (heterogeneous wall thickness) and enlargement of the pre-existing left upper-lobe cavity with irregular walls (Figure 2d). Cardiac troponin; the comprehensive cardiac enzyme panel; *Mycoplasma pneumoniae* antibodies; stool routine and fecal occult blood testing; sputum acid-fast bacillus smear; sputum *Mycobacterium tuberculosis* DNA; 1,3-*β*-D-glucan; Epstein–Barr virus DNA; and tumor markers were unremarkable; antigen screening for hepatitis A, B, and C viruses and HIV was negative.

After admission, the existing anti-tuberculosis regimen was continued, and empiric moxifloxacin 0.4 g IV once daily was added to broaden coverage for non-mycobacterial pathogens. Given concern for post–COVID-19 immune pneumonitis or an atypical-pathogen–related hyperinflammatory response, baricitinib was introduced for immunomodulation. Upon confirmation of a left-sided pleural air–fluid collection, a chest tube was placed for drainage. Nebulized N-acetylcysteine and salbutamol were maintained, with Tanreqing injection provided for symptomatic relief. Following these measures, chest pain improved markedly; however, cough and sputum production persisted, and the maximum temperature reached 39.5 °C. On February 7, 2023, flexible bronchoscopy showed blood-tinged BALF with patent airways. Brushings from the left main bronchus were submitted for bacteriology and cytology, and BALF was sent for culture, mNGS, rifampicin-resistance testing, and exfoliative cytology. On February 9, 2023, BALF-mNGS detected *Mycobacterium tuberculosis* (442 reads, relative abundance 12.98%) and a single read of *Orientia tsutsugamushi* (< 0.01%) (see Supplementary Figures 1–3). Real-time PCR for rifampicin resistance in BALF was negative. BALF cytology showed approximately 40% epithelial cells and 60% inflammatory cells (neutrophils 70%, lymphocytes 20%, histiocytes 10%) (Figure 2e). In the clinical context, coinfection with *O. tsutsugamushi* was strongly considered; serum IgM IFA was positive at a titer of 1:160 (Figure 2f). On February 21, 2023, the patient was discharged in stable condition with instructions to take medications regularly and to follow up at the local tuberculosis hospital. On March 1, under local guidance, doxycycline was discontinued while anti-tuberculosis therapy was continued. On June 2, 2023, outpatient CT showed a marked decrease in bilateral infectious foci with cavity shrinkage and areas of fibrosis; the apicoposterior-segment cavity of the left upper lobe had resolved, and most pleural effusion had been absorbed (Figure 2h), indicating a favorable response. N-acetylcysteine (600 mg, twice daily) was recommended, and addition of pirfenidone for antifibrotic therapy was advised; however, for personal reasons the patient declined pirfenidone, took N-acetylcysteine for one month, and then stopped it on his own. He continued regular sputum cultures and chest imaging at the local tuberculosis hospital and discontinued anti-tuberculosis drugs on October 26, 2023. On April 17, 2024, he re-presented to our clinic with chest tightness and dyspnea. CT demonstrated resolution of the prior upper-lobe cavities with bilateral focal reticular and linear opacities—predominantly in the right lower lobe (dorsal segment) and part of the left lower lobe—together with cavitary-like change and tractional distortion around the right hilum, consistent with fibrotic band formation after lesion repair (Figure 2i). Post-infectious pulmonary fibrosis was considered. Hospital admission for evaluation and treatment was recommended but declined. Long-term pirfenidone and home oxygen therapy were initiated: 200 mg three times daily initially, up-titrated over two weeks to a maintenance dose of 600 mg three times daily.

## 3 Discussion

This case illustrates a rare coinfection of cavitary pulmonary tuberculosis and scrub typhus caused by *Orientia tsutsugamushi*. Although conventional imaging, histopathology, and molecular assays rapidly established tuberculosis, the patient’s high-grade fever persisted despite guideline-concordant therapy. On admission, BALF real-time PCR for rifampicin resistance was negative, decreasing the likelihood of drug-resistant tuberculosis and suggesting an additional concomitant disease process. Reported instances of tuberculosis–scrub typhus coinfection are scarce but have been described in Southeast Asia and southern China, including cases of pulmonary tuberculosis with hemorrhagic fever with renal syndrome and scrub typhus, and tuberculous meningitis complicated by scrub typhus meningitis; in both, high-throughput sequencing and serology established the diagnosis and guided effective treatment (6, 7). Compared with prior reports, our case adds clarity by demonstrating that: (i) coinfection occurred in cavitary pulmonary tuberculosis—a phenotype prone to intense inflammation and secondary pathogens; (ii) early BALF-mNGS was pursued when the patient appeared unresponsive to anti-tuberculosis therapy, providing a practical trigger for broad-spectrum testing; and (iii) rapid defervescence followed organism-specific treatment, underscoring that timely recognition of dual pathogens can shorten the time to appropriate therapy and help limit downstream injury. mNGS is a hypothesis-free, broad-spectrum diagnostic approach that can survey viruses, bacteria, fungi, and parasites within a single assay (8). In this patient, bronchoalveolar lavage mNGS promptly indicated coexistence of *Mycobacterium tuberculosis* and *O. tsutsugamushi*, which was subsequently corroborated by serologic IgM testing. Prior studies show that mNGS outperforms conventional methods for pathogen detection in difficult-to-diagnose pulmonary infections and fever of unknown origin, and is particularly valuable for rapidly identifying rare or mixed infections (9–12). In this case, the low read abundance for *O. tsutsugamushi* on mNGS is likely multifactorial: (i) trace contamination during specimen collection or processing may generate low-level background signals; (ii) early antimicrobial therapy and host immune clearance can markedly reduce circulating or local pathogen nucleic-acid burden; and (iii) when infection is focal and the baseline bacterial load is low, the detected signal may approach the analytical limit of detection. Nevertheless, the totality of evidence—including clinical and epidemiologic features consistent with scrub typhus, a positive IgM serology, and rapid defervescence after targeted therapy—favors true infection over contamination. Given the inherent uncertainty of low-abundance reads, mNGS findings should be interpreted in conjunction with serology and other orthogonal modalities, while considering sampling chronology and treatment history, to minimize missed or incorrect diagnoses (13, 14).

After the diagnosis of cavitary pulmonary tuberculosis, a guideline-concordant regimen (isoniazid, rifampicin, ethambutol, and pyrazinamide) was initiated to control the primary lesions and reduce mycobacterial burden (15). Although rifampicin exhibits some *in vitro* and clinical activity against *Orientia tsutsugamushi*, its dose and dosing frequency in anti-tuberculosis therapy are optimized for the pharmacodynamics of *Mycobacterium tuberculosis* and may not sustain bactericidal intracellular concentrations against *O. tsutsugamushi* for a sufficient duration (16, 17). As an obligate intracellular pathogen with a relatively prolonged replication cycle, *O. tsutsugamushi* can persist and drive chronic inflammation if not suppressed with an adequate, targeted course (18). International guidelines and evidence syntheses list doxycycline or azithromycin as first-line agents for scrub typhus, whereas rifampicin is considered an alternative in selected scenarios with overall limited evidence (17). In this case, once mNGS and serology confirmed *O. tsutsugamushi*, doxycycline 0.1 g (100 mg) orally twice daily was added for 21 days on top of anti-tuberculosis therapy. The patient defervesced within 48 h with marked improvement in respiratory symptoms, mirroring the rapid clinical response to tetracyclines described in the literature (17). Doxycycline achieves favorable intracellular penetration, covers the pathogen’s replication window, and has been reported to inhibit matrix metalloproteinase activity, potentially limiting extracellular matrix degradation and lung tissue injury and thereby slowing post-inflammatory fibrotic remodeling (18–20). In patients with chronic tuberculosis lesions and/or impaired host defenses, dual pathogens may amplify inflammatory intensity, disease extent, and reparative responses, increasing the risk of parenchymal destruction and fibrosis (18, 21, 22). Scrub typhus can induce interstitial pneumonia and diffuse alveolar damage with upregulation of pro-fibrotic mediators, while tuberculosis foci maintain a low-grade inflammatory microenvironment; together, these processes may form a feed-forward loop that accelerates fibrosis (18, 21). For such complex infections, combining anti-tuberculosis and anti-*O. tsutsugamushi* therapy not only reduces pathogen load and dampens inflammation promptly but also creates a therapeutic window for subsequent antifibrotic interventions (22, 23). Despite microbiologic control in our patient, follow-up imaging documented post-infectious pulmonary fibrosis characterized by patchy reticulation and linear opacities, with exertional dyspnea requiring long-term low-flow oxygen (22). Given the lack of standardized treatment for post-infectious pulmonary fibrosis, current practice often extrapolates from idiopathic pulmonary fibrosis/progressive fibrosing ILD paradigms—pirfenidone or nintedanib—alongside pulmonary rehabilitation, long-term oxygen therapy, and nutritional support (23, 24). Although the patient initially declined pirfenidone, later adherence combined with home oxygen was associated with gradual symptomatic improvement. This trajectory underscores the importance of early recognition of coinfection and integrated pathogen-directed plus antifibrotic management to optimize long-term respiratory function and quality of life. Beyond scrub typhus, tuberculosis in Asia frequently coexists with other pathogens and carries important diagnostic and therapeutic implications. In Southeast Asia, a single-center study from Cambodia reported that among adults evaluated for respiratory infection, 33% of those with pulmonary tuberculosis had concomitant non-mycobacterial bacterial infections—predominantly *Klebsiella* and *Pseudomonas*—and coinfection was particularly common in patients with cavitary disease (25), underscoring the need to actively investigate secondary pathogens in cavitary tuberculosis. Viral–tuberculosis interactions also warrant attention: systematic reviews indicate measurable bidirectional effects between influenza and tuberculosis; during the COVID-19 period, tuberculosis–SARS-CoV-2 coinfection has been associated with increased disease severity and mortality, and clinicians must consider contraindications between antivirals and rifampicin (e.g., nirmatrelvir/ritonavir) (26, 27). Finally, post- tuberculosis structural lung disease in Asia confers susceptibility to chronic pulmonary aspergillosis (CPA); recent syntheses and multicenter surveys—several from Indonesia—identify CPA as a common yet under-recognized sequel of tuberculosis with substantial mortality, mandating vigilance for fungal coinfection and careful antifungal selection, particularly regarding rifampicin–triazole interactions (28–31). Taken together, these regional data support a systematic search for coinfections in Asian tuberculosis cases whenever persistent fever, cavitation, or discordant treatment responses are present.

Notably, this case occurred in a non-endemic area and without a typical outdoor exposure history, suggesting either a shifting geographic distribution of scrub typhus or the presence of under-recognized, cryptic transmission chains (32, 33). In China, the incidence and spatial footprint of scrub typhus have expanded substantially in recent years: the national annual incidence rose from 0.09 per 100,000 in 2006 to 1.60 per 100,000 in 2016, and further to 1.93 per 100,000 in 2018, with case counts increasing exponentially (32, 34, 35). Spatially, affected rural townships increased from 422 to 4,083 and urban towns from 194 to 942 between 2006 and 2016; by 2011–2016, cases had been reported across all 31 provinces (32). Provinces previously regarded as non-endemic—such as Shandong and Heilongjiang—have reported cases since 1986, with sporadic occurrences in cities and non-traditional rural settings as well (33, 35, 36). These shifts likely reflect multiple drivers, including climate change, northward expansion of suitable habitats for chiggers and reservoir hosts, population mobility, and improved diagnostic capacity (32, 33, 35). Clinically, this means that—even in non-endemic regions—persistent fever under appropriate anti-tuberculosis therapy with unrevealing routine tests should prompt consideration of scrub typhus and other vector-borne diseases, with early deployment of broad-spectrum assays such as mNGS. From a public health perspective, surveillance networks for vector-borne infections should be strengthened and expanded—particularly as climate change enlarges potential habitats—and front-line clinicians’ awareness and diagnostic proficiency regarding scrub typhus should be enhanced to facilitate early recognition of uncommon coinfections.

## 4 Conclusion

In regions non-endemic for scrub typhus, persistent fever in patients with tuberculosis despite guideline-concordant therapy and unrevealing routine tests should prompt early consideration of coinfection and a comprehensive pathogen work-up, including mNGS where available. Coinfection with *Mycobacterium tuberculosis* and *Orientia tsutsugamushi* may increase diagnostic and therapeutic complexity and heighten the risk of post-infectious pulmonary fibrosis; accordingly, pathogen control should be paired with lung-function preservation and longitudinal follow-up. This case provides preliminary evidence that concomitant infection with *M. tuberculosis* and *O. tsutsugamushi* may contribute to the development of pulmonary fibrosis; however, a single case report is insufficient to establish causality. Rigorous mechanistic investigations and well-designed controlled studies are warranted to elucidate the underlying pathophysiology and to define optimal strategies for patient selection, pharmacotherapy, and monitoring.

## Data Availability

The original contributions presented in the study are included in the article/Supplementary material, further inquiries can be directed to the corresponding author.

## References

[1] Xu G Walker DH Jupiter D Melby PC Arcari CM. A review of the global epidemiology of scrub typhus. PLoS Negl Trop Dis. (2017) 11:e0006062. doi: 10.1371/journal.pntd.0006062, PMID: 29099844 PMC5687757

[2] Perumalla SK Paul S Abhilash KPP Gunasekaran K Rose W Mahasampath G . Eschar and IgM ELISA in the diagnosis of scrub typhus. Indian J Med Microbiol. (2019) 37:113–5. doi: 10.4103/0255-0857.264495, PMID: 31424021

[3] Rose W Rajan RJ Punnen A Ghosh U. Distribution of eschar in pediatric scrub typhus. J Trop Pediatr. (2016) 62:415–20. doi: 10.1093/tropej/fmw027 27122479

[4] Peter JV Sudarsan TI Prakash JAJ Varghese GM. Severe scrub typhus infection: clinical features, diagnostic challenges and management. World J Crit Care Med. (2015) 4:244–50. doi: 10.5492/wjccm.v4.i3.244, PMID: 26261776 PMC4524821

[5] Trajman A Campbell JR Kunor T Ruslami R Amanullah F Behr MA . Tuberculosis. Lancet. (2025) 405:850–66. doi: 10.1016/S0140-6736(24)02479-6, PMID: 40057344

[6] Huang H Kong Y Yin H Yang Z Ren T Zhang Y. A case of pulmonary tuberculosis patient complicated with hemorrhagic fever with renal syndrome and scrub typhus in Yunnan, China: a case report. BMC Infect Dis. (2023) 23:631. doi: 10.1186/s12879-023-08416-4, PMID: 37752443 PMC10523743

[7] Tan M Zou H Yang M Liu A. *Orientia tsutsugamushi* meningitis in a patient with tuberculous meningitis complications: a case report. Front Med. (2025) 12:1591785. doi: 10.3389/fmed.2025.1591785, PMID: 40636361 PMC12237661

[8] Le Van N Pham Van C Nguyen Dang M Dao Van T Le T Do Q Vu Hoang H . Clinical features, laboratory characteristics and prognostic factors of severity in patients with Rickettsiaceae at two military hospitals, northern Vietnam. Infect Drug Resist. (2020) 13:2129–38. doi: 10.2147/IDR.S253540, PMID: 32753908 PMC7351622

[9] Gao Q Li L Su T Liu J Chen L Yi Y . A single-center, retrospective study of hospitalized patients with lower respiratory tract infections: clinical assessment of metagenomic next-generation sequencing and identification of risk factors in patients. Respir Res. (2024) 25:250. doi: 10.1186/s12931-024-02887-y, PMID: 38902783 PMC11191188

[10] Yin Y Zhu P Guo Y Li Y Chen H Liu J . Enhancing lower respiratory tract infection diagnosis: implementation and clinical assessment of multiplex PCR-based and hybrid capture-based targeted next-generation sequencing. EBioMedicine. (2024) 107:105307. doi: 10.1016/j.ebiom.2024.105307, PMID: 39226681 PMC11403251

[11] Chen H Huang Q Wu W Wang Z Wang W Liu Y . Assessment and clinical utility of metagenomic next-generation sequencing for suspected lower respiratory tract infections. Eur J Med Res. (2024) 29:213. doi: 10.1186/s40001-024-01806-7, PMID: 38561853 PMC10983704

[12] Shen H Liu T Shen M Zhang Y Chen W Chen H . Utilizing metagenomic next-generation sequencing for diagnosis and lung microbiome probing of pediatric pneumonia through bronchoalveolar lavage fluid in pediatric intensive care unit: results from a large real-world cohort. Front Cell Infect Microbiol. (2023) 13:1200806. doi: 10.3389/fcimb.2023.1200806, PMID: 37655299 PMC10466250

[13] You Y Ni YM Shi G. Diagnostic accuracy of metagenomic next-generation sequencing in pulmonary tuberculosis: a systematic review and meta-analysis. Syst Rev. (2024) 13:317. doi: 10.1186/s13643-024-02733-8, PMID: 39731100 PMC11674177

[14] Tan JK Servellita V Stryke D Kelly E Streithorst J Sumimoto N . Laboratory validation of a clinical metagenomic next-generation sequencing assay for respiratory virus detection and discovery. Nat Commun. (2024) 15:9016. doi: 10.1038/s41467-024-51470-y, PMID: 39532844 PMC11558004

[15] Nahid P Dorman SE Alipanah N Barry PM Brozek JL Cattamanchi A . Official American Thoracic Society/Centers for Disease Control and Prevention/Infectious Diseases Society of America clinical practice guidelines: treatment of drug-susceptible tuberculosis. Clin Infect Dis. (2016) 63:e147–95. doi: 10.1093/cid/ciw376, PMID: 27516382 PMC6590850

[16] Watt G Kantipong P Jongsakul K Watcharapichat P Phulsuksombati D Strickman D. Doxycycline and rifampicin for mild scrub-typhus infections in northern Thailand: a randomised trial. Lancet. (2000) 356:1057–61. doi: 10.1016/S0140-6736(00)02728-8, PMID: 11009140

[17] El Sayed I Liu Q Wee I Hine P. Antibiotics for treating scrub typhus. Cochrane Database Syst Rev. (2018) 9:CD002150. doi: 10.1002/14651858.CD002150.pub2, PMID: 30246875 PMC6485465

[18] Trent B Fisher J Soong L. Scrub typhus pathogenesis: innate immune response and lung injury during *Orientia tsutsugamushi* infection. Front Microbiol. (2019) 10:2065. doi: 10.3389/fmicb.2019.02065, PMID: 31555249 PMC6742975

[19] Fujita M Ye Q Ouchi H Harada E Inoshima I Kuwano K . Doxycycline attenuated pulmonary fibrosis induced by bleomycin in mice. Antimicrob Agents Chemother. (2006) 50:739–43. doi: 10.1128/AAC.50.2.739-743.2006, PMID: 16436734 PMC1366885

[20] Fujita H Sakamoto N Ishimatsu Y Kakugawa T Hara S Hara A . Effects of doxycycline on production of growth factors and matrix metalloproteinases in pulmonary fibrosis. Respiration. (2011) 81:420–30. doi: 10.1159/000324080, PMID: 21502778

[21] Hsu YH Chen HI. Pulmonary pathology in patients associated with scrub typhus. Pathology. (2008) 40:268–71. doi: 10.1080/00313020801911488, PMID: 18428046

[22] Migliori GB Marx FM Ambrosino N Zampogna E Schaaf HS van der Zalm MM . Clinical standards for the assessment, management and rehabilitation of post-TB lung disease. Int J Tuberc Lung Dis. (2021) 25:797–813. doi: 10.5588/ijtld.21.0425, PMID: 34615577 PMC8504493

[23] Raghu G Remy-Jardin M Richeldi L Thomson CC Inoue Y Johkoh T . Idiopathic pulmonary fibrosis (an update) and progressive pulmonary fibrosis in adults: an official ATS/ERS/JRS/ALAT clinical practice guideline. Am J Respir Crit Care Med. (2022) 205:e18–47. doi: 10.1164/rccm.202202-0399ST, PMID: 35486072 PMC9851481

[24] Flaherty KR Wells AU Cottin V Devaraj A Walsh SLF Inoue Y . Nintedanib in progressive fibrosing interstitial lung diseases. N Engl J Med. (2019) 381:1718–27. doi: 10.1056/NEJMoa1908681, PMID: 31566307

[25] Attia EF Pho Y Nhem S Sok C By B Phann D . Tuberculosis and other bacterial co-infection in Cambodia: a single center retrospective cross-sectional study. BMC Pulm Med. (2019) 19:60. doi: 10.1186/s12890-019-0828-4, PMID: 30866909 PMC6417204

[26] Walaza S Cohen C Tempia S Moyes J Nguweneza A Madhi SA . Influenza and tuberculosis co-infection: a systematic review. Influenza Other Respir Viruses. (2020) 14:77–91. doi: 10.1111/irv.12670, PMID: 31568678 PMC6928059

[27] Wang Y Chen Y Gu L Lou L Zhang J Zhang K. The clinical characteristics and risk factors for severe COVID-19 in patients with COVID-19 and tuberculosis coinfection. Front Microbiol. (2022) 13:1061879. doi: 10.3389/fmicb.2022.1061879, PMID: 36619998 PMC9817148

[28] Madden A Ofori SK Budu M Sisay E Dooley B Murray MB. A systematic review of chronic pulmonary aspergillosis among patients treated for pulmonary tuberculosis. Clin Infect Dis. (2025):ciaf150. doi: 10.1093/cid/ciaf150 PMC12596383 40117217

[29] Rozaliyani A Setianingrum F Isbaniah F Agustin H Handayani RRD Syahrir R . A silent threat in post-tuberculosis patients: chronic pulmonary aspergillosis survey in multiple regions of Indonesia (I-CHROME study). J Fungi (Basel). (2025) 11:329. doi: 10.3390/jof11050329, PMID: 40422663 PMC12112450

[30] Rozaliyani A Rosianawati H Handayani D Agustin H Zaini J Syam R . Chronic pulmonary aspergillosis in post tuberculosis patients in Indonesia and the role of LDBio aspergillus ICT as part of the diagnosis scheme. J Fungi (Basel). (2020) 6:318. doi: 10.3390/jof6040318 33260909 PMC7712371

[31] Lewis R Niazi-Ali S McIvor A Kanj SS Maertens J Bassetti M . Triazole antifungal drug interactions-practical considerations for excellent prescribing. J Antimicrob Chemother. (2024) 79:1203–17. doi: 10.1093/jac/dkae103, PMID: 38629250 PMC11977760

[32] Li Z Xin H Sun J Lai S Zeng L Zheng C . Epidemiologic changes of scrub typhus in China, 1952–2016. Emerg Infect Dis. (2020) 26:1091–101. doi: 10.3201/eid2606.191168, PMID: 32441637 PMC7258452

[33] Yao H Wang Y Mi X Sun Y Liu K Li X . The scrub typhus in mainland China: spatiotemporal expansion and risk prediction underpinned by complex factors. Emerg Microbes Infect. (2019) 8:909–19. doi: 10.1080/22221751.2019.1631719, PMID: 31233387 PMC6598543

[34] Luo Y Zhang L Lv H Zhu C Ai L Qi Y . How meteorological factors impacting on scrub typhus incidences in the main epidemic areas of 10 provinces, China, 2006–2018. Front Public Health. (2022) 10:992555. doi: 10.3389/fpubh.2022.992555, PMID: 36339235 PMC9628745

[35] Yue Y Ren D Liu X Wang Y Liu Q Li G. Spatio-temporal patterns of scrub typhus in mainland China, 2006–2017. PLoS Negl Trop Dis. (2019) 13:e0007916. doi: 10.1371/journal.pntd.0007916, PMID: 31790406 PMC6917297

[36] Yang LP Liang SY Wang XJ Li XJ Wu YL Ma W. Burden of disease measured by disability-adjusted life years and a disease forecasting time series model of scrub typhus in Laiwu, China. PLoS Negl Trop Dis. (2015) 9:e3420. doi: 10.1371/journal.pntd.0003420, PMID: 25569248 PMC4288724

