# Supplementary material for: Cavitary pulmonary tuberculosis with Orientia tsutsugamushi coinfection in a non-endemic region: a case report
Source: Front Med (Lausanne). 2025 Dec 3;12:1692918. doi: 10.3389/fmed.2025.1692918 (PMC12708244; doi:10.3389/fmed.2025.1692918)
Supplement: Supplementary file 1 [file Supplementary_file_1.pdf]

Supplementary Material

1 Supplementary Data

Table 1. Laboratory Results by Date

Key laboratory tests summarized chronologically. Abbreviations: ABG, arterial blood gas; AFB, acid-fast bacillus; BALF, bronchoalveolar lavage fluid; IFA, immunofluorescence assay.

Date	Specimen / Module	Test / Index	Result	Units	Reference range	Interpretation / Notes
2023-02-04	Arterial blood gas (2 L/min O2)	Base excess	+4.6	mmol/L		
2023-02-04	Arterial blood gas (2 L/min O2)	HCO3−	29.8	mmol/L		
2023-02-04	Arterial blood gas (2 L/min O2)	PaCO2	46	mmHg		
2023-02-04	Arterial blood gas (2 L/min O2)	PaO2	106	mmHg		
2023-02-04	Arterial blood gas (2 L/min O2)	PaO2/FiO2	365.5	mmHg		Preserved oxygenation
2023-02-04	Arterial blood gas (2 L/min O2)	SaO2	98	%		
2023-02-04	Arterial blood gas (2 L/min O2)	pH	7.42	—		

2023-02-04	Chemistry	Albumin	34.6	g/L		Low
2023-02-04	Chemistry	Calcium	2.03	mmol/L		Low
2023-02-04	Chemistry	Glucose	6.82	mmol/L		Mildly high
2023-02-04	Chemistry	Phosphorus	0.83	mmol/L		Low
2023-02-04	Chemistry	Total protein	55.4	g/L		Low
2023-02-04	Chemistry	Urea	3.04	mmol/L		Mild abnormality
2023-02-04	Complete blood count	Hematocrit	41.9	%		Low
2023-02-04	Complete blood count	Hemoglobin	134.0	g/L		Low-normal
2023-02-04	Complete blood count	Lymphocytes (relative)	13.3	%		Low
2023-02-04	Complete blood count	Monocytes (absolute)	1.23	×10^9/L		High

2023-02-04	Complete blood count	Monocytes (relative)	11.1	%		High
2023-02-04	Complete blood count	Neutrophils (absolute)	8.22	×10^9/L		High
2023-02-04	Complete blood count	Nucleated RBCs	0.1	%		Present
2023-02-04	Complete blood count	RDW-SD	50.40	fL		High
2023-02-04	Complete blood count	Red blood cells	4.40	×10^12/L		Slightly low
2023-02-04	Complete blood count	White blood cells	11.09	×10^9/L		High
2023-02-04	Enzymes	Lactate dehydrogenase (LDH)	264	U/L		High
2023-02-04	Enzymes	α-Hydroxybutyrate dehydrogenase (α-HBDH)	213	U/L		High
2023-02-04	Inflammation/Coagulation	C-reactive protein (CRP)	25.59	mg/L		High
2023-02-04	Inflammation/Coagulation	Fibrinogen	6.17	g/L		High

2023-02-04	Lymphocyte subsets	CD3+ T cells	1,152	cells/µL		
2023-02-04	Lymphocyte subsets	CD4+ T cells	636	cells/µL		
2023-02-04	Lymphocyte subsets	CD4/CD8 ratio	1.41	—		Normal ratio
2023-02-04	Lymphocyte subsets	CD8+ T cells	452	cells/µL		
2023-02-04	Screening / Panels	Multiple items	Negative / unremarkable			Cardiac troponin & cardiac enzyme panel; Mycoplasma pneumoniae antibodies; Stool routine & fecal occult blood; Sputum AFB smear; Sputum Mycobacterium tuberculosis DNA; 1,3-β-D-glucan; Epstein–Barr virus DNA; Tumor markers; Hepatitis A/B/C antigen screening;

						HIV antigen screening
2023-02-09	BALF - Cytology (differential)	Epithelial cells	≈40%	%		
2023-02-09	BALF - Cytology (differential)	Inflammatory cells	≈60%	%		Neutrophils 70%; Lymphocytes 20%; Histiocytes 10%
2023-02-09	BALF - RT-PCR	Rifampicin resistance	Negative			
2023-02-09	BALF - mNGS	Mycobacterium tuberculosis	442 reads			Relative abundance 12.98%
2023-02-09	BALF - mNGS	Orientia tsutsugamushi	1 read			<0.01% relative abundance
2023-02-09	Serology	O. tsutsugamushi IgM IFA	1:160			Positive

Note: Units are shown as reported. "Interpretation / Notes" reflects clinical context (e.g., High/Low). If journal requires emphasis of abnormal values, consider bolding and adding ↑/↓ during copyediting.

Figures 1-3 metagenomic next-generation sequencing (mNGS) results

二，检测结果

测序质量

质控信息			
	总序列数	Q20	Q30
合格标准	>10,000,000	>90%	>85%
DNA+RNA	42,063,709	97.22%	96.02%

注：以上表格说明此次检测测序数据质量合格，结果可信。

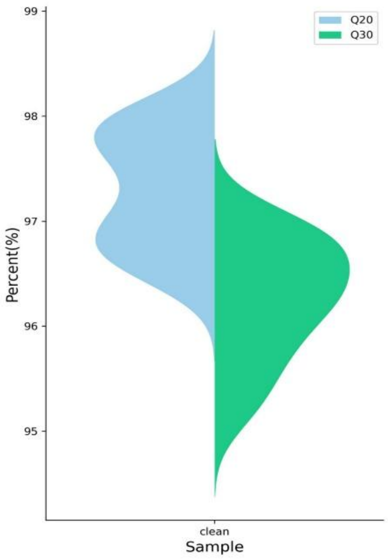

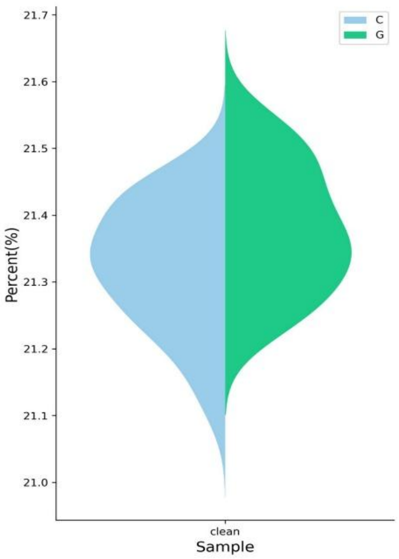

注：上图分别表示 clean reads 碱基质量及 GC 含量分布情况。左图中蓝色表示 Q20 的分布情况，绿色表示 Q30 分布情况。右图蓝色表示 C 碱基含量的分布情况，绿色表示 G 碱基含量的分布情况。

结果综述

关注微生物
结核分枝杆菌复合群，恙虫病东方体

疑似或人体共生微生物群
—

Q-mNGS™检测结果供参考，建议结合患者症状和其它临床检测结果进一步确认感染情况。

人源背景

Host index（人源指数）	Host index 在同类标本中的分布
32442.20，高于 62.64%的同类样本	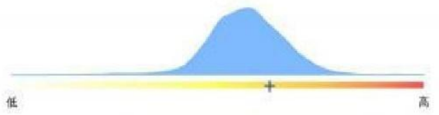

通过内标分子的测定值，计算标本人源核酸背景（数值越高，标本人源背景越高）。

详细结果列表

1.细菌（Bacteria）

属（Genus）				种（Species）		
类型	名称	序列数	相对丰度%	名称	序列数	相对丰度%
未检出						

2.分枝杆菌（Mycobacteria）

属（Genus）			种（Species）		
名称	序列数	相对丰度%	名称	序列数	相对丰度%

分枝杆菌属 Mycobacterium	442	＜12.98 %	结核分枝杆菌复合群 Mycobacterium tuberculosis complex	442	12.98%

3.支原体/衣原体/立克次体（Mycoplasma/Chlamydia/Rickettsia）

属（Genus）			种（Species）		
名称	序列数	相对丰度%	名称	序列数	相对丰度%
东方体属 *Orientia*	1	＜0.01 %	恙虫病东方体 *Orientia tsutsugamushi*	1	＜0.01%

4. 真菌（Fungi）

属（Genus）			种（Species）		
名称	序列数	相对丰度%	名称	序列数	相对丰度%
未检出					

5. DNA 病毒（DNA viruses）

属（Genus）			种（Species）/ 亚型（Subtype）		
名称	序列数	相对丰度%	名称	序列数	相对丰度%
未检出					

6. RNA 病毒（RNA viruses）

属（Genus）			种（Species）/ 亚型（Subtype）		
名称	序列数	相对丰度%	名称	序列数	相对丰度%
未检出					
